# Ptomaines

**Published:** 1889-07

**Authors:** John A. Miller

**Affiliations:** Professor of Medical Chemistry and Toxicology at the Medical Department of Niagara University; Chemical Laboratory, Niagara University


### Original Lectures.

### PTOMAINES.

By JOHN A. MILLER, M. A., Ph. D.,

Professor of Medical Chemistry and Toxicology at the Medical Department of Niagara University. Synopsis of a lecture delivered March 21st.

Ptomaines are chemical compounds, of a basic character, formed during the putrefaction of organic matter. The name, which was given to these compounds by the Italian chemist, Selmi, is derived from the Greek word "ptoma," meaning cadaver, to which the syllable "ine" has been added as an index to their chemical character.

In mineral chemistry, we recognize such substances as soda, potassa, and ammonia as bases. They are compounds which having alkaline solutions give the power of changing red litmus paper to blue. They possess, however, another property which characterizes them as bases, *i. e.*, the power to form salts with acids.

Organic chemistry presents the largest variety of bases, a large number of which contain nitrogen, and it is to a class of these compounds, called "*amines*," that I specially wish to call your attention.

You remember that marsh-gas has the formula $CH_4$, also that we are able to replace one or all of the hydrogen atoms by any of the halogens, as chlorine, bromine, etc. If, now, we treat methyl chloride ($CH_3$ Cl.) with alcoholic ammonia, we obtain the hydrochloric acid salt of a base called *methylamine* ($CH_3$ $NH_2$ H Cl). We can replace still another atom of hydrogen, introducing another amidogen ($NH_2$) group, and obtain a compound called methyldiamine [$CH_2$ $(NH_2)_2$]. These compounds give us a clew to the chemical character of the ptomaines, *i. e.*, they are bases, but such as contain nitrogen as an essential part of their basic character.

The ptomaines are obtained from putrefying organic substances by various methods, dependent upon their solubility in various mediums. The character of the ptomaine isolated will depend upon the degree of putrefaction, upon the fact whether during decay there has been a limited or unlimited supply of oxygen, and upon the character of the bacteria at work. We naturally expect to find each variety of bacteria producing its own particular ptomaine. This does not seem an unreasonable belief, as the ptomaines are merely "transition products through which matter passes while being transformed, by the activity of bacterial life, from the organic to the inorganic state;" the very complex molecule being broken up into less complex ones, and so the process of chemical subdivision going on until the simple final products, carbonic acid, ammonia, and water result.

Recognizing, then, that microörganisms are the cause whereby an abnormal condition called disease may be produced in the system, the question naturally arises:

How do these organisms act so as to produce this particular abnormal condition called disease?

The contagious nature of apoplectiform anthrax was first demonstrated by Gerlach in 1845. Four years later, Pollender discovered numerous rod-like microörganisms in the blood of animals afflicted with this disease. Bollinger believed that these organisms caused a deoxidation of the blood, which was the producing cause of the symptoms and death. Virchow, however, reported a number of cases in which the blood of animals that had died from anthrax contained no bacilli, or contained them only in very limited numbers. Joffroy, on the other hand, inoculated healthy animals and found that they died from the effects of this disease before any bacilli appeared in the blood. Bollinger's theory was not entirely abandoned, however, until Nencki proved by a series of experiments that the physiological oxidation was in no way diminished, and consequently there could be no deoxidation of the blood.

Numerous theories were then offered to explain the action of bacteria in producing disease, but they all soon passed away into the history of theories.

The theory which is accepted to-day is, that the bacteria produce a chemical poison by splitting up pre-existing complex organic compounds in the body. These poisons are the ptomaines, and from pure cultures of bacillus anthracis Hoffa has succeeded in isolating a particular ptomaine which, when injected subcutaneously, produced the symptoms of the disease followed by death.

Prof. Brieger, of Berlin, has succeeded in isolating from slightly impure cultures of the *tetanus microbe* four poisonous substances, *tetanine*, *tetanotoxine*, *spasmotoxine*, and a fourth unnamed. From the amputated arm of a tetanus patient Brieger isolated *tetanine*, which was identical in its physiological action and chemical reactions with that obtained from cultures of the tetanus microbe. This compound, *tetanine*, when injected into animals, produces the characteristic, though not *all* the symptoms, of tetanus. The remaining three produce paralysis and convulsions.

From pure cultures of coma bacillus Brieger isolated several ptomaines, two of which he considers to be the specific product of coma bacillus. The first one appears to be trimethylenediamine ($C_3 H_8 N_2$). Judging from its physiological action, it appears to be identical with a certain basic compound which has been isolated from choleraic bodies. " It causes violent convulsions and tremor of the muscles." The second is unnamed, and its chemical composition unknown. When injected subcutaneously, it produced a paralysis-like, lethargic condition. Respiration and the heart's action became slower, the temperature was lowered, and death resulted in from twelve to twenty-four hours. In some cases, bloody stools were passed. It is to be hoped that further investigation will clear up many dark points in regard to these compounds.

Typhotoxine is the ptomaine which Brieger isolated from cultures of typhoid bacillus. He considers it to be the specific poison of typhoid fever, but as it causes no increase of temperature, it is very probable that future investigation will prove that more than one ptomaine is elaborated within the system. The investigations of Vaughan, Novy, Sirotinin and others seem to point to this belief; though they have not yet succeeded in isolating other ptomaines. After the injection of typhotoxine into mice and guinea-pigs, the following symptoms were observed: "First, slight salivation, with increased respiration; the animals lose control over the muscles of the trunk and extremities, and fall down helpless upon their sides. The pupils become strongly dilated, and cease to react to light; the salivation becomes more profuse; the rate of heart-beat and of respiration gradually decreases, and death follows in from one to two days. Throughout the course of these symptoms the animals have frequent diarrhœic evacuations, but at no time are convulsions present. On post mortem the heart is found to be contracted in systole, the lungs are strongly hyperemic, the other internal organs pale, the intestines firmly contracted, and their walls pale."

Bourget draws the following conclusions from his investigations of puerperal fever:

"1. In puerperal fever the urine contains highly poisonous bases. 2. The toxicity of the urine is most marked when the symptoms of the disease are most grave, and diminishes as the symptoms abate. 3. The ptomaines obtained from the urine prove fatal when injected into frogs and guinea-pigs. 4. Toxic bases, resembling those obtained from the urine, were extracted from the viscera of a woman who had died of puerperal fever."

The last ptomaine or toxic principle which I desirel to call your attention to, is the one isolated by Prof. Vaughan, of the Michigan University, from milk and cheese, and to which he has given the name—tyrotoxicon. From the investigations of Vaughan and the application of the knowledge thus obtained to his medical practice, Dr. Vaughan is of the belief that tyrotoxicon is the cause of many cases of cholera infantum. "As this poison appears very rapidly and abundantly in milk under favorable conditions, and furthermore as the symptoms produced by tyrotoxicon and the pathological changes observed on post mortem are identical with those occasioned by this disease," this conclusion appears to be very well grounded.

By stopping the use of cow's milk during the illness of patients afflicted with this disease, Vaughan has obtained excellent results.

Much work still remains to be done in this field of the ptomaines; they must be isolated and identified; their physiological action more carefully studied; and, finally, efforts must be made to ascertain what drugs act as prophylactics and antidotes.

CHEMICAL LABORATORY, NIAGARA UNIVERSITY.

